# Transarterial chemoembolization combined with lenvatinib plus tislelizumab for unresectable hepatocellular carcinoma: a multicenter cohort study

**DOI:** 10.3389/fimmu.2024.1449663

**Published:** 2024-10-01

**Authors:** Yushan Zhao, Shuwei Wen, YaoQing Xue, Zhijun Dang, ZhiYu Nan, Dong Wang, Xiao Li, Duiping Feng, Yi Chen

**Affiliations:** ^1^ Shanxi Province Cancer Hospital/Shanxi Hospital Affiliated to Cancer Hospital, Chinese Academy of Medical Sciences/Cancer Hospital Affiliated to Shanxi Medical University, Shanxi, China; ^2^ Department of Oncology Intervention, National Cancer Center, Beijing, China; ^3^ Department of Oncology and Vascular Intervention, First Hospital of Shanxi Medical University, Taiyuan, Shanxi, China; ^4^ Department of Oncology and Vascular Intervention, Shanxi Provincial Clinical Research Center for Interventional Medicine, Taiyuan, China

**Keywords:** TACE, lenvatinib, tislelizumab, hepatocellular carcinoma, overall survival

## Abstract

**Objective:**

Comparing the efficacy of transarterial chemoembolization (TACE) combined with lenvatinib plus tislelizumab (TLT) with TACE combined with lenvatinib (TL) for unresectable hepatocellular carcinoma, particularly in determining which patients can benefit more from the TLT treatment.

**Methods:**

From March 2021 to September 2023, a total of 169 patients from three centers were included in this study, with 103 patients receiving TLT and 66 patients receiving TL. The Kaplan-Meier method was utilized to evaluate the cumulative overall survival (OS) and progression-free survival (PFS) between the two groups and were assessed using the log-rank test. Subgroup analysis on tumor number, maximum tumor diameter, presence of portal vein thrombosis, AFP level, and Child-Pugh class were conducted.

**Results:**

The median OS was 26 months in the TLT group, and 20 months in the TL group. The median PFS was 14 months in the TLT group and 9 months in the TL group. The Kaplan-Meier curve demonstrated a significantly superior OS and PFS in the TLT group compared to the TL group. Subgroup analysis showed that for patients with a maximum tumor diameter greater than 7 cm, AFP > 400 ng/ml and accompanied by portal vein tumor thrombus, and Child-Pugh class A, there was a statistically significant difference in OS between TLT and TL groups.

**Conclusions:**

OS and PFS were significantly improved in patients who received TLT compared to those who received TL, patients with a largest tumor diameter greater than 7 cm, AFP > 400 ng/ml, Child-Pugh class A and PVTT appeared to derive more benefit.

## Introduction

Hepatocellular carcinoma (HCC) is the most common pathological type of primary liver cancer and is also the main cause of tumor-related deaths (1). Treatment methods for hepatocellular carcinoma include surgical resection, ablative therapy, transarterial chemoembolization (TACE), and systemic therapy (2). Systemic therapy has made rapid progress in recent years, with the application of tyrosine kinase inhibitors (TKI), anti-vascular endothelial growth factor (anti-VEGF) agents, programmed death 1 (PD-1) inhibitors and programmed death ligand 1 (PD-L1) inhibitors in clinical (3–9). A non-inferiority study conducted in 2018 demonstrated similar efficacy between lenvatinib (a tyrosine kinase inhibitor) and sorafenib in the treatment of advanced HCC (3). Therefore, lenvatinib has been approved as a first-line therapy and has achieved relatively satisfactory outcomes. In the IMbrave150 trial, the combination of atezolizumab (a PD-L1 inhibitor) and bevacizumab (an anti-VEGF antibody) showed superior overall survival and progression-free survival outcomes compared to sorafenib in patients with unresectable HCC (5). A study of lenvatinib plus pembrolizumab (a PD-1 inhibitor) in patients with unresectable hepatocellular carcinoma showed that lenvatinib plus pembrolizumab has promising antitumor activity (10).

However, the prognosis of advanced HCC is still poor. In order to improve the therapeutic efficacy, some researchers have started using combined local treatment with systemic therapy to treat advanced HCC (11–14). A phase III randomized clinical trial showed that combining TACE and lenvatinib is safer and more effective treatment for patients with advanced HCC compared to lenvatinib monotherapy (15). Some studies have shown that the combination of TACE with lenvatinib and PD-1 inhibitors can significantly improve the survival of HCC patients (16–19). These studies confirm the definite efficacy of local treatments combined with systemic treatment for advanced HCC. However, most previous studies have focused on the combination of TACE with lenvatinib and PD-1 inhibitors, with few studies specifically focusing on a single PD-1 inhibitor.

Tislelizumab has been to be used in HCC patients in recent years. However, additional evidence is required to establish whether TACE combined with lenvatinib and tislelizumab offers superior outcomes compared to TACE combined with lenvatinib. Moreover, detailed subgroup analyses are still needed to identify patients who may benefit more from the triple therapy.

Therefore, this study aims to further validate the efficacy of TACE combined with lenvatinib plus tislelizumab by conducting a multicenter cohort analysis of advanced HCC patients who received TACE combined with lenvatinib with or without tislelizumab.

## Methods

The Ethics Board of the First Hospital of Shanxi Medical University, Shanxi Cancer hospital and National Cancer Center approved this retrospective study. Since the study was conducted retrospectively and involved anonymous data, the requirement for informed consent from each patient was waived.

### Patients

The detailed medical records of hepatocellular carcinoma patients who received TACE plus lenvatinib with or without tislelizumab were collected at the First Hospital of Shanxi Medical University, Shanxi Cancer Hospital and National Cancer Center from March 2021 to September 2023. All patients included in the study were diagnosed through image-guided needle biopsy or non-invasive criteria. The non-invasive diagnostic criteria consist of three key factors. Firstly, patients had a documented history of liver cirrhosis. Secondly, the tumor size of ≥1cm in diameter. Lastly, there should be noticeable arterial enhancement followed by subsequent washout in the venous or delayed phase, as observed through dynamic magnetic resonance imaging or enhanced computed tomography (20).

The inclusion criteria for this study were as follows: 1) at least one measurable lesion, 2) patients unable to undergo surgical resection, 3) patients with no prior history of systemic treatment including immunotherapy or TKI therapy, 4) patients with a Child-Pugh classification of A or B, and 5) patients with an Eastern Cooperative Oncology Group Performance Status (ECOG-PS) score of 0-1. The exclusion criteria included patients with autoimmune diseases, acute infectious lesions, and the concurrent presence of other malignancies.

### TACE procedure

TACE procedures are performed by doctors with over 10 years of experience in interventional radiology. The TACE procedure is conducted under local anesthesia with guidance from digital subtraction angiography. After puncture, a 5F catheter is advanced to perform hepatic artery angiography to identify the tumor’s supplying arteries. To identify all tumor-supplying arteries, it may be necessary to perform diaphragmatic artery angiography, mesenteric artery angiography, and intra-thoracic artery angiography. Once the tumor-supplying arteries were identified, a microcatheter was used for super-selective catheterization of the tumor supplying arteries. After confirming the angiographic findings, embolization was performed. Iodized oil (Lipiodol; Guerbet, France) mixed with epirubicin (50 mg/m^2^) or drug-eluting microspheres mixed with epirubicin (40-80 mg) were used for embolization (21). If necessary, supplemental embolization can be performed using gelatin sponge particles or additional microspheres. Repeat angiography was performed after embolization. Patients should undergo a follow-up examination with enhanced CT or dynamic enhanced MRI at 4-6 weeks to evaluate the degree of lesion necrosis, the presence of new lesions, liver function status, and physical condition to assess the need for repeat TACE.

### System therapy protocol

Patients were administered lenvatinib orally within 2-5 days after TACE at a daily dose of 12 mg if they had a body weight greater than 60 kg, or 8 mg for those with a body weight was less than 60 kg. Some patients also received an intravenous dose of 200 mg of tislelizumab on day 1 of each 21-day therapy cycle.

### Follow-up

Follow-up visits were scheduled approximately one month after the initiation of TACE. During each visit, patients underwent various tests, including chest CT, abdomen multiphase CT or MRI, and total bilirubin (TBIL), aspartate aminotransferase (AST), alanine aminotransferase (ALT), albumin (ALB), prothrombin time (PT), and AFP level evaluation. Overall survival was defined as the period from the first TACE procedure until either death from any cause or the last follow-up (22). Progression-free survival was defined as the period from the first TACE until either tumor progression or the last follow-up. Tumor progression was considered to have occurred if there was a 25% increase in baseline tumor size, transient deterioration of liver function to Child-Pugh C, presence of macrovascular invasion, the development of extrahepatic metastasis (23).

### Statistical analysis

Categorical variables were presented as frequencies (percentages) and compared using Fisher’s exact test or the chi-square test, as appropriate. The Kaplan-Meier method was employed to assess the cumulative overall survival and progression-free survival. The differences between two groups were evaluated using the log-rank test. Univariate and multivariate analyses were performed using Cox regression to assess OS. To further analyses the differences in treatment efficacy between the two treatment modalities, subgroup analysis on tumor number, maximum tumor diameter, presence of portal vein thrombosis, Child-Pugh class and AFP level were conducted. All statistical analyses were performed using IBM SPSS Statistics (version 23.0). A two-sided test of significance was conducted, with a *P*-value < 0.05 considered statistically significant.

## Results

In our study, a total of 169 patients were eventually included, among which 66 patients received TACE combined with lenvatinib (TL), and 103 patients received TACE combined with lenvatinib plus tislelizumab (TLT). The baseline characteristics of patients between two groups were shown in **Table 1**. There were no statistically differences between the two groups in terms of age, gender, largest tumor diameter, number of tumors, HBV infection status, Child-Pugh class, AFP levels, TBIL levels and ALB levels, presence of portal vein thrombosis (PVTT), prognostic nutritional index (PNI), extrahepatic metastasis and BCLC period (*P>*0.05).

**Table 1:** Baseline data of patients between two groups were compared.

Patients		TL Group (n=66)	TLT Group (n=103)	*P*
**Age (years)**				**0.377**
≤70	144	54 (81.8%)	90 (87.4%)	
>70	25	12 (18.2%)	13 (12.6%)	
**Sex**				**0.078**
Female	31	7 (10.6%)	24 (23.3%)	
Male	137	59 (89.4%)	78 (75.7%)	
**Largest diameter of the tumor (cm)**				**1.000**
≤7	64	25 (37.9%)	39 (37.9%)	
>7	105	41 (62.1%)	64 (62.1%)	
**Tumor number**				**0.302**
≤ 3	49	16 (24.27%)	33 (32.0%)	
> 3	120	50 (75.8%)	70 (68.0%)	
**HBV**				**0.080**
NO	46	23 (34.8%)	23 (22.3%)	
Yes	123	43 (65.2%)	80 (77.7%)	
**Child-Pugh class**				**0.828**
A	147	57 (83.82%)	90 (82.57%)	
B	30	11 (16.18%)	19 (17.43%)	
**AFP (ng/mL)**				**0.525**
≤400	73	26 (39.4%)	47 (45.6%)	
>400	96	40 (60.6%)	56 (54.4%)	
**TBIL (µmol/L)**				**0.533**
≤32	140	53 (80.3%)	87(84.5%)	
>32	29	13 (19.7%)	16 (15.5%)	
**ALB(g/L)**				**0.136**
≤40	59	28(42.4%)	31 (30.1%)	
>40	110	38 (57.6%)	72 (69.9%)	
**PVTT**				**0.752**
NO	91	37 (56.1%)	54(52.4%)	
Yes	78	29 (43.9%)	49 (47.6%)	
**PNI**				**0.467**
≤44.5	94	39 (59.1%)	55(53.4%)	
>44.5	75	27(40.9%)	48(46.6%)	
**Extrahepatic Metastasis**				**0.563**
NO	52	22 (33.3%)	30 (29.1%)	
Yes	119	44 (66.7%)	73 (70.9%)	
**BCLC Period**				**0.978**
B	28	11 (16.7%)	17(16.5%)	
C	141	55(83.3%)	86 (83.5%)	

HBV, Hepatitis B virus; AFP, serum α-fetoprotein; TBIL, Total bilirubin; ALB, albumin; PVTT, Portal Vein Tumor Thrombosis; BCLC, Barcelona Clinic Liver Cancer; PNI, Prognostic Nutritional Index.

### Overall survival

**Figure 1:**
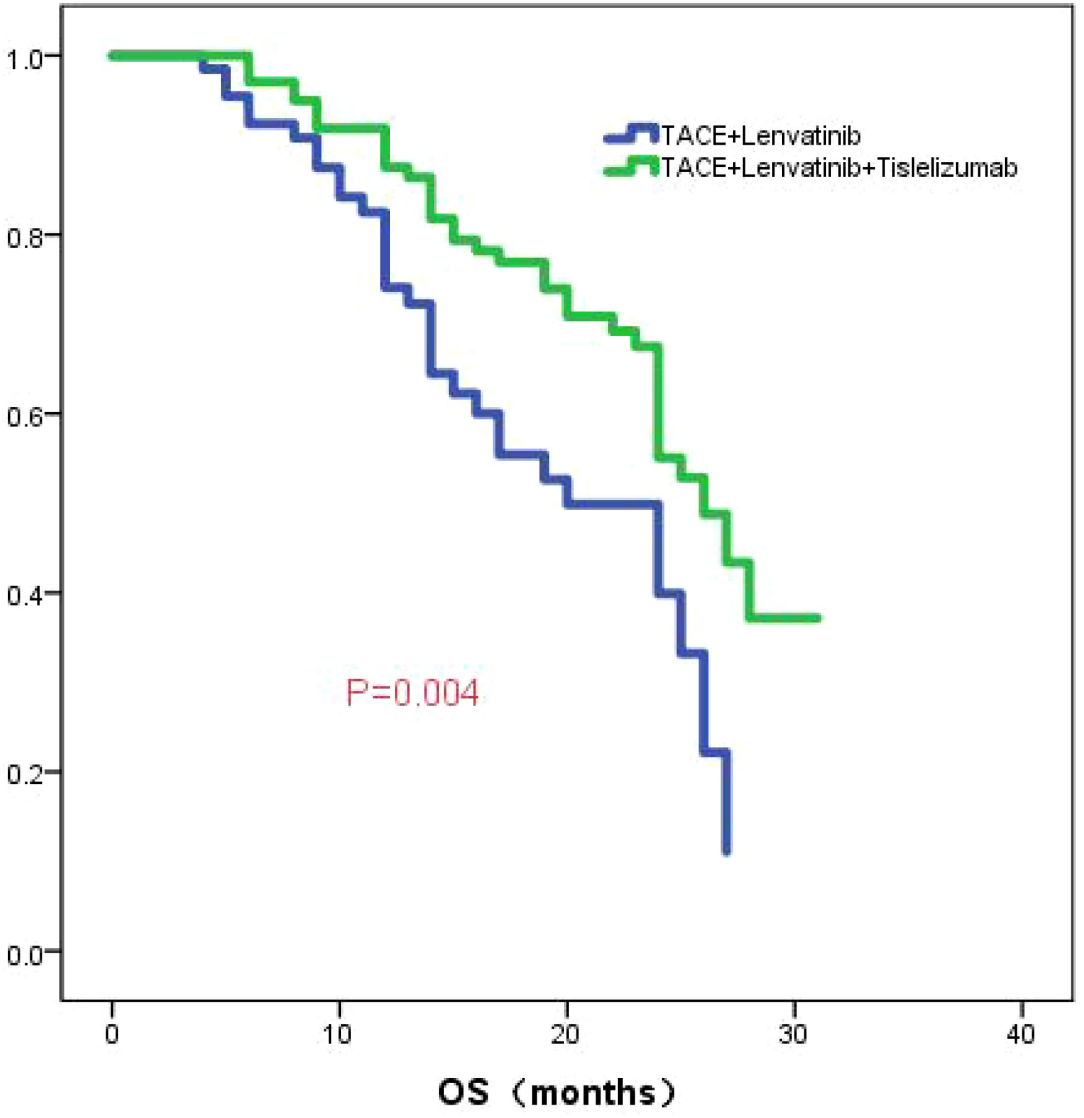
The KM curve displays the overall survival between TLT group and TL group.

The median follow-up time was 23.0 months (95%CI:20.9-25.1). The median OS in the TL group was 20.0 months (95% CI: 13.6-26.4), while the median OS in the TLT group was 26.0 months (95% CI: 22.5-29.5). The Kaplan-Meier curve demonstrated a significantly superior OS in the TLT group compared to the TL group (*P*=0.004; **Figure 1**). Based on univariate and multivariate Cox regression analyses, largest diameter of the tumor, the type of treatment, the TBIL level, and the PNI score have been identified as independent prognostic factors of OS (refer to **Table 2**).

**Table 2:** Univariate and multivariate analyses for OS in patients receiving TL and TLT.

Variables (OS)	Univariate Analysis			Multivariate Analysis		
	HR	95% CI	*P-value*	HR	95% CI	*P-value*
Age (years)						
≤70	1.428	0.749-2.725	0.279	–	–	–
>70						
Sex						
Female	1.444	0.754-2.765	0.268	–	–	–
Male						
Largest diameter of the tumor (cm)						
≤7	2.642	1.581-4.417	<0.001*	2.490	1.415-4.382	0.002
>7						
Tumor number						
≤ 3	1.128	0.681-1.870	0.640	–	–	–
> 3						
HBV						
NO	1.193	0.697-2.042	0.520	–	–	–
Yes						
Child-Pugh class						
A	3.132	1.818-5.398	<0.001*	1.053	0.517-2.145	0.886
B						
AFP (ng/mL)						
≤400	1.966	1.217-3.175	0.006	1.396	0.827-2.355	0.211
>400						
TBIL (µmol/L)						
≤32	2.474	1.401-4.369	0.002	2.305	1.166-4.559	0.016
>32						
Extrahepatic Metastasis						
NO	0.754	0.461-1.235	0.262	–	–	–
Yes						
PVTT						
NO	1.373	0.857-2.201	0.188	–	–	–
Yes						
Treatment						
TACE+Lenvatinib	0.511	0.316-0.825	0.006	0.508	0.310-0.830	0.007
TACE+Lenvatinib+Tislelizumab						
PNI						
≤44.5	0.367	0.220-0.611	<0.001*	0.434	0.250-0.753	0.003
>44.5						

### Progression-free survival

**Figure 2:**
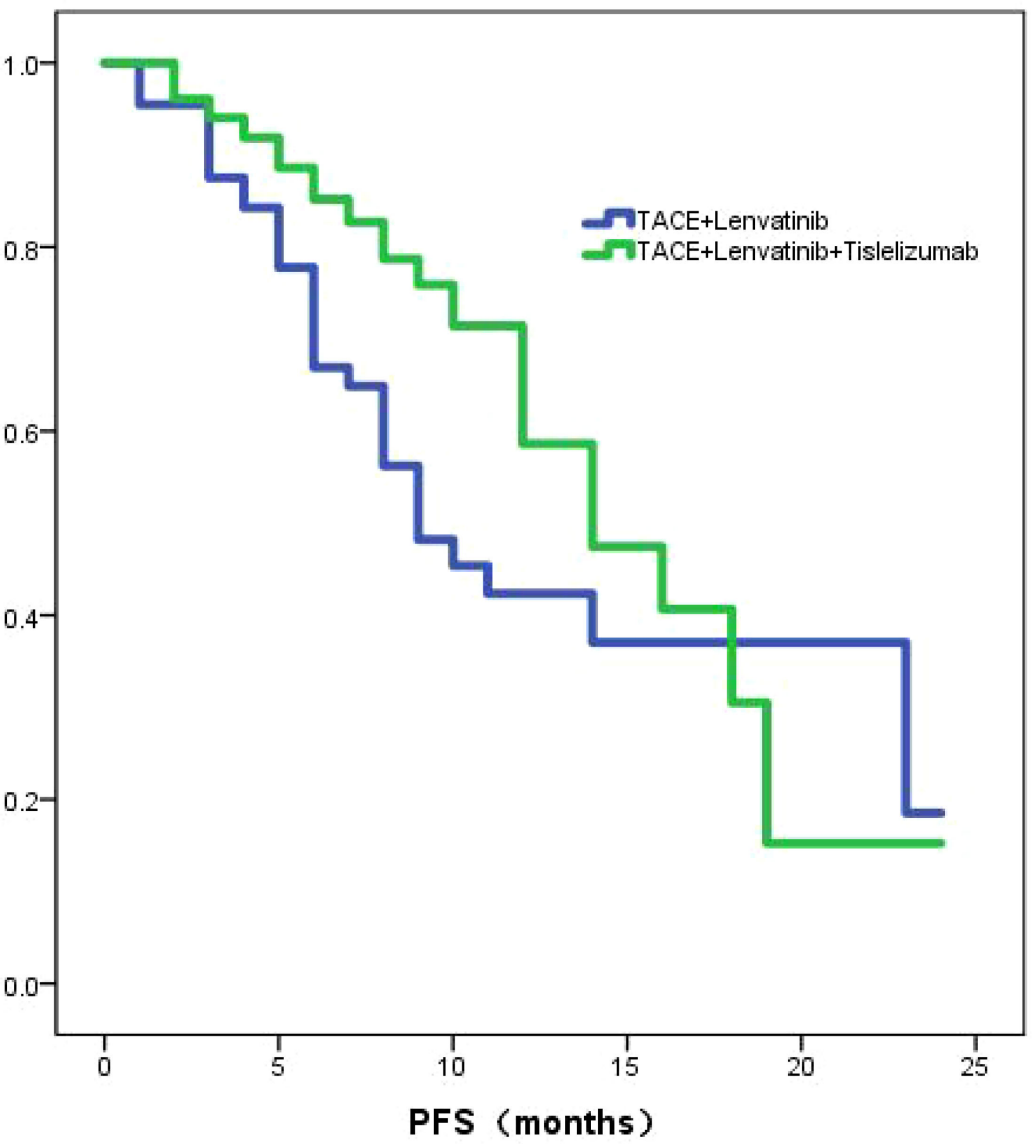
The KM curve shows the PFS between TLT group and TL group.

The TL group exhibited a median PFS of 9.0 months (95% CI: 5.9-12.0), whereas the TLT group demonstrated a longer median PFS of 14.0 months (95% CI:10.9-17.1). The Kaplan-Meier curves revealed a statistically significant difference (*P*=0.039; **Figure 2**) in PFS between the two groups. Through univariate and multivariate Cox regression analysis, the type of treatment, Child-Pugh class, and PNI were identified as independent prognostic factors for PFS (**Table 3**).

**Table 3:** Univariate and multivariate analyses to assess the prognostic factors for PFS.

Variables (PFS)	Univariate Analysis			Multivariate Analysis		
	HR	95% CI	*P-value*	HR	95% CI	*P-value*
Age (years)						
≤70	1.443	0.753-2.765	0.269	–	–	–
>70						
Sex						
Female	1.464	0.746-2.870	0.267	–	–	–
Male						
Largest diameter of the tumor (cm)						
≤7	2.198	1.276-3.785	0.005	1.799	0.996-3.249	0.052
>7						
Tumor number						
≤ 3	1.063	0.639-1.769	0.814	–	–	–
> 3						
HBV						
NO	1.233	0.721-2.108	0.445	–	–	–
Yes						
Child-Pugh						
A	5.290	2.951-9.481	<0.001*	4.352	2.379-7.959	<0.001*
B						
AFP (ng/mL)						
≤400	1.727	1.070-2.787	0.025	1.357	0.802-2.295	0.255
>400						
TBIL (µmol/L)						
≤32	1.726	0.976-3.053	0.061	–	–	–
>32						
Extrahepatic Metastasis						
NO	0.982	0.600-1.608	0.943	–	–	–
Yes						
PVTT						
NO	1.241	0.774-1.990	0.370	–	–	–
Yes						
Treatment						
TACE+Lenvatinib	0.617	0.383-0.993	0.047	0.513	0.313-0.841	0.008
TACE+Lenvatinib+Tislelizumab						
PNI						
≤44.5	0.392	0.235-0.653	<0.001*	0.476	0.281-0.805	0.006
>44.5						

### Objective response rate

The overall response rates evaluated using mRECIST criteria in the two treatment groups were compared in **Table 2**. In the TLT group, there were 5 cases of complete response and 63 cases of partial response. In the TL group, there were 2 cases of complete response and 29 cases of partial response. According to the mRECIST criteria, the objective response rate (ORR) was 66.1% in the TLT group and 46.9% in the TL group (*P*=0.014, **Table 4**).

**Table 4:** The comparison of ORRs between the two treatment modalities.

Variable	TLT group(n, %)	TL group(n, %)	P value
Complete response	5(4.9%)	2(3.0%)	
Partial response	63(61.2%)	29(43.9%)	
Stable disease	30(29.1%)	30(45.5%)	
Progressive disease	5(4.8%)	5(7.6%)	
Objective response	68(66.1%)	31(46.9%)	0.014

### Subgroup analysis results

**Figure 3:**
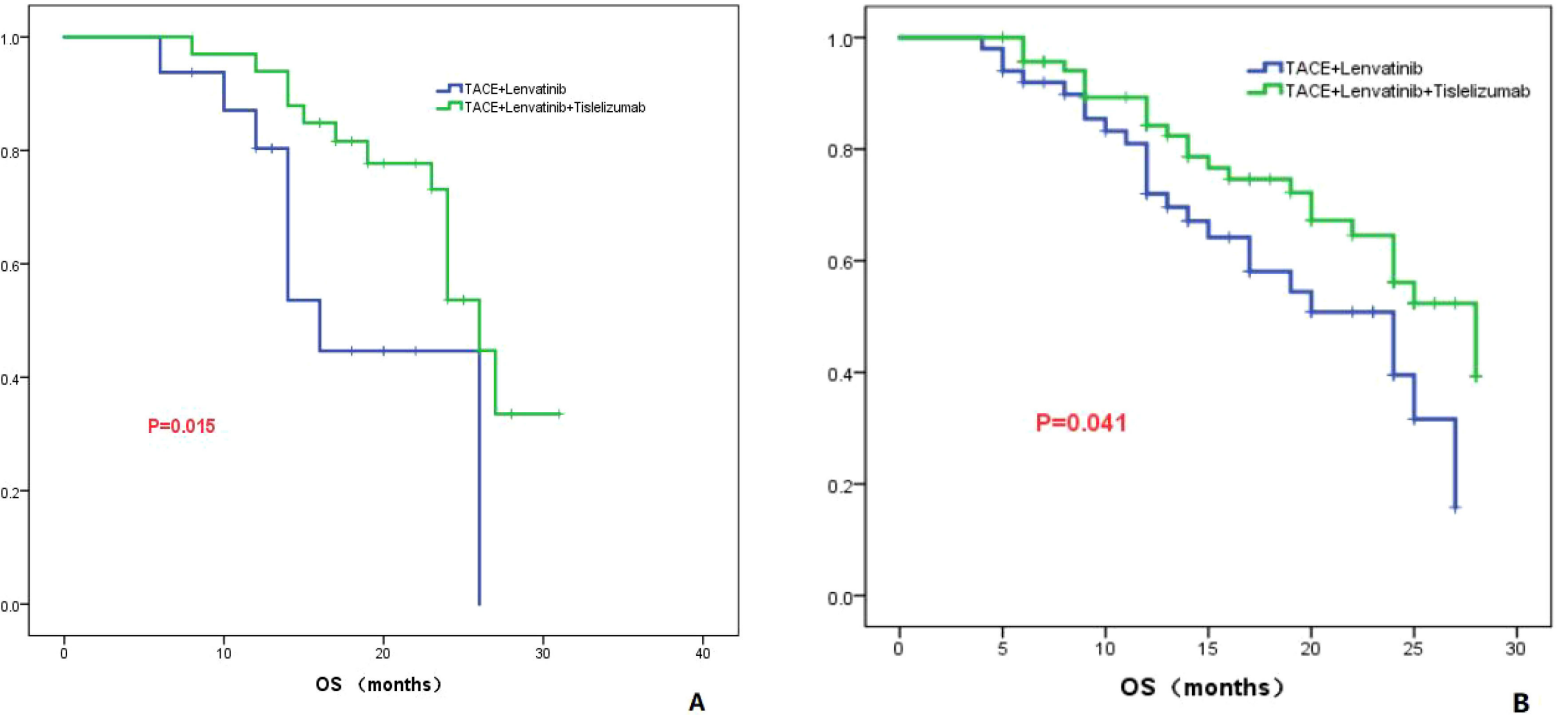
The KM curves demonstrate the comparison between TLT and TL group in OS for tumor numbers less than 3 **(A)** or greater than 3 **(B)**.

Tumor number: According to the number of tumors, all patients were divided into two groups: one group with a tumor count of ≤3 (Group 1), and the other group with a tumor count >3 (Group 2). In Group 1, the median OS for the TL group was 16.0 months (95% CI: 12.8-19.2), while the median OS for the TLT group was 26.0 months (95% CI: 23.5-28.5). The difference in OS between the two groups was statistically significant (*P*=0.015; **Figure 3A**). In Group 2, the TL group had a median OS of 24.0 months (95% CI: 16.9-31.0), whereas the TLT group had a median OS of 28.0 months (95% CI: 22.0-33.9). The difference in OS between the two groups was statistically significant (*P*=0.041; **Figure 3B**).

#### Largest diameter of the tumor

**Figure 4:**
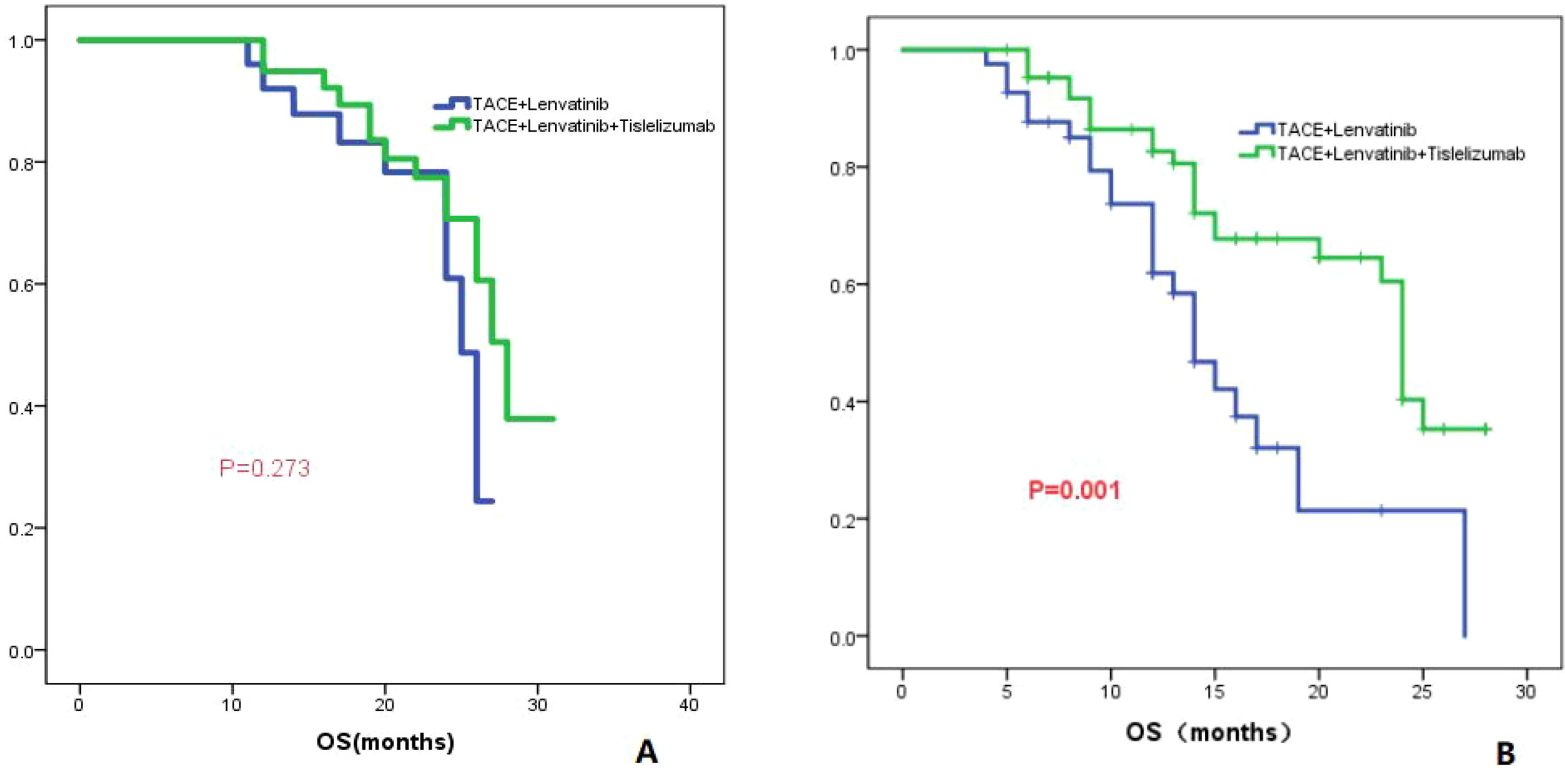
The KM curves display the comparison of OS between the TLT and TL groups for tumors with a maximum diameter of less than or equal to 7cm **(A)** and greater than 7cm **(B)**.

The patients were divided into two groups based on whether the largest tumor diameter was greater than 7 cm or not. Group 1 consisted of patients with a tumor diameter less than or equal to 7 cm, while group 2 consisted of patients with a tumor diameter greater than 7 cm. In Group 1, the TL group had a median OS of 25.0 months (95% CI: 23.3-26.6), while the TLT group had a median OS of 28.0 months (95% CI: 25.4-30.6). There was no significant difference in OS between the two groups (*P*=0.273; **Figure 4A**). In Group 2, the TL group had a median OS of 14.0 months (95% CI: 11.8-16.2), whereas the TLT group had a median OS of 24.0 months (95% CI: 23.1-24.9). There was a significant difference in OS between the two groups (*P=*0.001; **Figure 4B**).

#### PVTT

**Figure 5:**
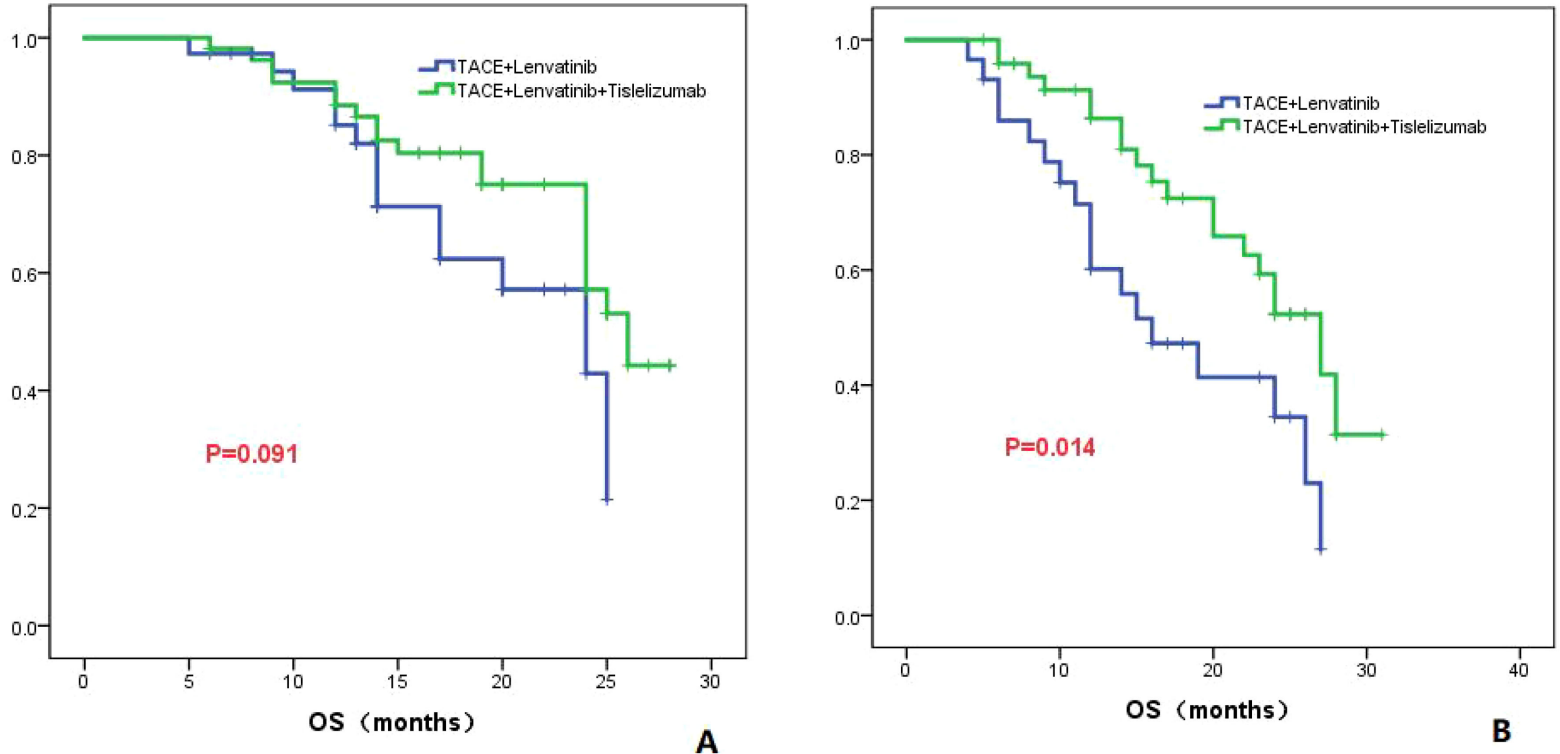
The KM curves elucidate the variation in OS between the TLT and TL groups for patients either without **(A)** or with PVTT **(B)**.

The patients were divided into two groups based on the presence or absence of PVTT, Group 1 consisted of patients without PVTT, while Group 2 consisted of patients with PVTT. In Group 1, the TL group demonstrated a median OS of 24.0 months (95% CI: 16.1-31.9), while the TLT group exhibited a median OS of 26.0 months (95% CI: 22.7-29.3). No statistically significant difference in OS was observed between the two groups (*P*=0.091; **Figure 5A**). In Group 2, the TL group displayed a median OS of 16.0 months (95% CI: 9.3-22.7), whereas the TLT group exhibited a longer median OS of 27.0 months (95% CI: 21.8-32.2). A significant difference in OS was observed between the two groups (*P*=0.014; **Figure 5B**).

#### Child-Pugh class

**Figure 6:**
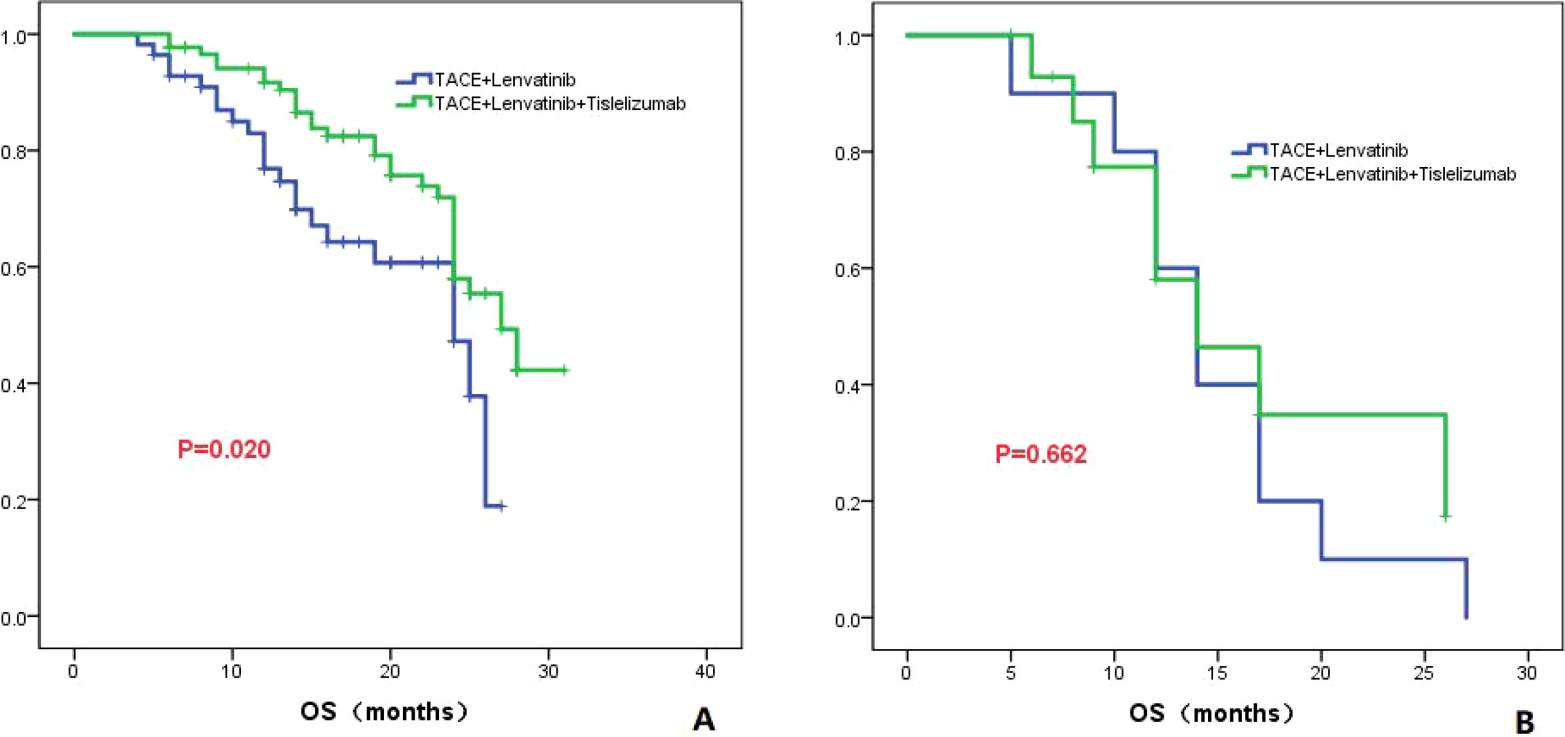
The KM curves highlight the variance in OS between the TLT and TL groups in patients classified as Child-Pugh grade A **(A)** and B **(B)**.

In patients classified as Child-Pugh class A, the TL group had a median OS of 24.0 months (95% CI: 18.7-29.3), while the TLT group had a higher median OS of 27.0 months (95% CI: 23.3-30.7). There was a statistically significant difference in OS between the two groups (*P*=0.020; **Figure 6A**). However, in patients classified as Child-Pugh class B, both the TL group and the TLT group had a similar median OS of 14.0 months. There was no significant difference in OS between the two groups (*P*=0.662; **Figure 6B**).

#### AFP level

**Figure 7:**
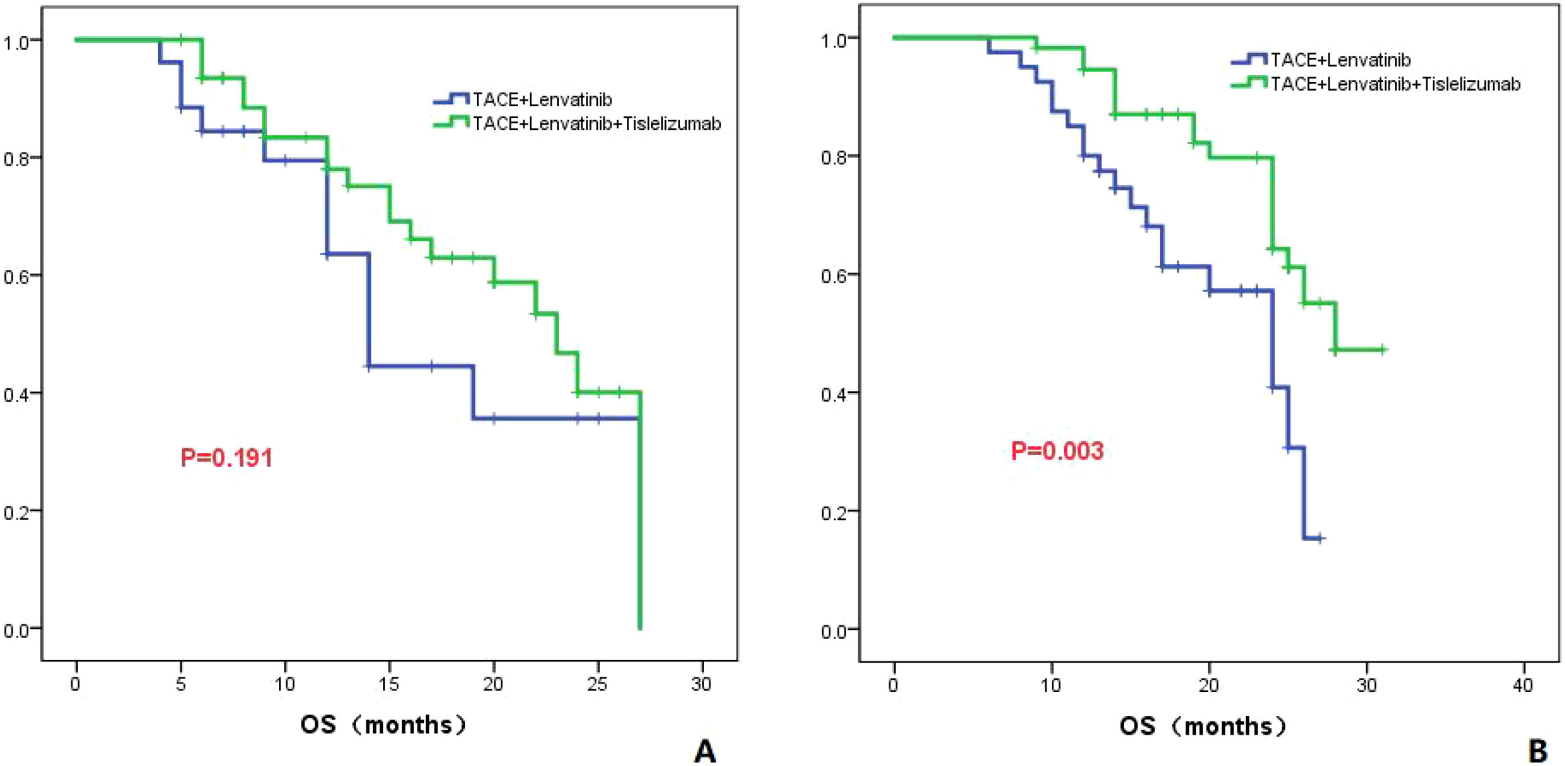
The KM curves astutely display the variance in OS between the TLT and TL groups within patients presenting AFP levels of ≤400ng/ml **(A)** or >400ng/ml **(B)**.

In patients with AFP less than 400 ng/ml, the median OS was 14.0 months (95%CI: 11.5-16.4) in the TL group and 23.0 months (95%CI: 18.7-27.3) in the TLT group. There was no significant difference in OS between the two groups (*P*=0.191; **Figure 7A**). In patients with AFP greater than 400 ng/ml, the median OS was 24.0 months (95%CI: 18.4-29.6) in the TL group and 28 months in the TLT group. There was a significant difference in OS between the two groups (*P*=0.003; **Figure 7B**).

In the TLT group, the common adverse events (AEs) were abdominal pain, nausea, fatigue, hypertension, and fever. In the TL group, the common AEs were abdominal pain, nausea, fever, decreased appetite, and hypertension. Among the patients in the TLT group, 22 individuals (21.3%) experienced grade 3-4 AEs, while in the TL group, 11 out of 66 patients (16.7%) reported grade 3-4 AEs (**Table 5**).

**Table 5:** Treatment-Related AEs in TLT and TL group.

AEs	Any Grade NO.(%)		Grade 3-5 NO.(%)	
	TLT group(n=103)	TL group(n=66)	TLT group(n=103)	TL group(n=66)
Abdominal pain	60(58.2%)	32(48.5%)	5(4.9%)	2(3.0%)
Nausea	48(46.6%)	29(43.9%)	4(3.9%)	3(4.5%)
Fatigue	45(43.7%)	20(30.3%)	1(0.9%)	0(0%)
Hypertension	40(38.8%)	23(34.8%)	5(4.9%)	3(4.5%)
Fever	35(40.0%)	28(42.4%)	0(0%)	0(0%)
Decreased appetite	26(25.2%)	25(37.8%)	3(2.9%)	2(3.0%)
Hand-foot skin reaction	12(11.6%)	9(13.6%)	2(1.9%)	1(1.5%)
Abnormal liver function	23(22.3%)	13(19.7%)	2(1.9%)	0(0%)

## Discussion

Tislelizumab is a monoclonal antibody that exhibits a strong affinity and binding specificity for PD-1. It has demonstrated remarkable effectiveness and a well-tolerated safety profile in patients with various solid tumors (24–26). In the open-label, global, multiregional phase 3 RATIONALE-301 randomized clinical trial, tislelizumab exhibited a noninferior overall survival benefit compared to sorafenib, along with a higher objective response rate and more sustained responses and showed a longer median progression-free survival (27).

To further investigate the role of tislelizumab treatmenting HCC, we compared the efficacy of TACE combined with lenvatinib versus TACE combined with lenvatinib plus tislelizumab. The study demonstrated that the OS in the TLT group was significantly superior to the TL group, with median OS of 26 and 20 months, respectively, showing a statistically significant difference (*P*=0.004). The PFS in the TLT group was also significantly better than the TL group, with a noticeable difference between the two groups (*P*=0.039). The ORR in the TLT group was 66.1%, whereas it was 46.9% in the TL group. The TLT group was significantly superior to the TL group(*P*=0.014). The study had preliminarily demonstrated that the combination of TACE with lenvatinib plus tislelizumab significantly improves OS, PFS and ORR compared to TACE combined with lenvatinib, it has also shown good safety profile. These results are consistent with previous studies focusing on other PD-1 inhibitors, providing further evidence for the treatment of HCC (28, 29).

To further clarify the advantages of TACE combined with lenvatinib and tislelizumab in the treatment of HCC, subgroup analyses of patients based on tumor number, maximum tumor diameter, presence of PVTT, AFP level, and Child-Pugh classification were conducted. For patients with a tumor maximum diameter less than or equal to 7 cm, there was no significant difference in OS between the TLT group and the TL group (*P*=0.273). However, for patients with a tumor maximum diameter greater than 7 cm, the TLT group showed a significantly improved OS compared to the TL group, with a statistically significant difference (*P*=0.001). These findings suggest that for patients with a tumor maximum diameter greater than 7 cm, the combination with tislelizumab can significantly prolong survival. For patients without PVTT, there was no significant difference in OS between the TLT and TL groups (*P*=0.091). However, for patients with PVTT, the TLT group showed significantly longer OS compared to the TL group (*P*=0.014), indicating that the addition of tislelizumab benefits patients with PVTT. Previous studies have consistently shown that the combination of PD-1 inhibition is effective and well-tolerated for treating patients with PVTT, and the results of this study align with these findings (30). The study also revealed that in patients with AFP greater than 400 ng/ml, the TLT group had significantly better overall survival compared to the TL group. However, in patients with AFP less than 400 ng/ml, there was no significant difference in OS between the two groups. The findings suggest that for HCC patients with relatively high AFP level, TLT is more beneficial than TL.

In this study, it was also observed that there was a significant difference in OS between the TLT and TL groups in patients with Child-Pugh A (*P*=0.020). However, no difference in OS was found between the two groups in patients with Child-Pugh B (*P*=0.662). This suggests that the Child-Pugh classification is crucial in determining the efficacy of combination therapy, as patients with poor liver function may not benefit from additional combined treatments. It also highlights the importance of selecting patients with relatively good scores on Child-Pugh grade for TACE combined with lenvatinib and tislelizumab therapy.

This is the first study comparing the efficacy of TACE plus lenvatinib with or without tislelizumab. The study has shown that TLT can significantly improve OS and PFS compared to TL. Subgroup analysis revealed that patients with the maximum tumor diameter greater than 7 cm, AFP > 400ng/ml and the presence of PVTT may benefit more from TLT treatment. It was also noted that the TLT therapy had a more pronounced effect in patients with a better Child-Pugh score. Compared to previous studies (28–31), this study not only found that patients with PVTT benefit more from the triple therapy, but also discovered that patients with larger tumor diameters and higher AFP levels benefit more from the triple therapy.

This study had some limitations. Firstly, it is a retrospective study with a limited number of cases, which may lead to selection bias and further confirmation is needed with a larger sample size. Secondly, majority of patients in this study were HBV-infected (123/169), which may not be representative of patients with HCC of other etiologies. Lastly, patients may have received other treatment modalities after disease progression, which could potentially confound the analysis of OS. In our upcoming research, we will further analyze the mechanisms by which TKI combined with PD-1 enhances efficacy and identify biomarkers that predict prognosis.

## Conclusion

TACE combined with lenvatinib plus tislelizumab was significantly better than TACE combined with lenvatinib in OS, PFS and ORR for unresectable HCC. Further subgroup analysis reveals that TLT treatment provides greater benefits for patients with tumor diameters greater than 7 cm, accompanied by PVTT, AFP levels greater than 400ng/ml, and being in the Child-Pugh A stage.

## Data Availability

The raw data supporting the conclusions of this article will be made available by the authors, without undue reservation.
